# Dynamic responses of gut microbiota to agricultural and wildfire ash: insights from different amphibian developmental stages

**DOI:** 10.3389/fmicb.2025.1598446

**Published:** 2025-08-13

**Authors:** Qing Tong, Ming-da Xu, Qiu-ru Fan, Yue-liang Pan, Xin-zhou Long, Wen-jing Dong, Li-yong Cui, Zhi-wen Luo

**Affiliations:** ^1^School of Biology and Agriculture, Jiamusi University, Jiamusi, China; ^2^Jiamusi Branch of Heilongjiang Academy of Forestry Sciences, Jiamusi, China

**Keywords:** *Rana dybowskii*, gut microbiota, wildfire ash, rice straw, survival

## Abstract

Combustion by-products—specifically wildfire ash and rice-straw ash—are emerging contaminants in freshwater ecosystems. However, their impacts on amphibian survival and gut microbiota across various developmental stages remains largely unclear, thereby limiting evidence-based conservation strategies in fire-affected habitats. This study evaluated the effects of artificial water (control, C) and aqueous extracts of ash (AEAs) derived from wildfire ash (W) and rice straw ash (S) on the survival and gut (G) microbiota of *Rana dybowskii* tadpoles (T) and adult frogs (F). Exposure to wildfire ash significantly reduced tadpole survival compared to rice straw ash, whereas no significant differences were observed in adult frogs. Alpha diversity of the gut microbiota differed significantly among tadpole groups but not among adult groups. Bray-Curtis and weighted UniFrac analyses revealed significant differences in the gut microbiota of adult frogs and tadpoles across different treatment groups. Linear discriminant analysis effect size (LEfSe) identified a significant enrichment of specific bacterial genera across treatment groups. BugBase analysis indicated that in the TCG, TSG, and TWG groups, notable variations in the TCG, TSG, and TWG groups, there were notable differences in Forms-Biofilms and Potentially-Pathogenic, while in the FCG, FSG, and FWG groups, significant differences were observed in Aerobic, Gram-Positive, Potentially-Pathogenic, and Stress-Tolerant. These findings suggest that wildfire ash exhibits greater toxicity than rice straw ash to both life stages of *R. dybowskii*, with tadpoles being more vulnerable. By elucidating the link between ash-derived pollutants and amphibian gut health, this study underscores the growing threat of routine straw burning and intensifying wildfires to global freshwater biodiversity and advocates for ash-specific mitigation measures and microbiota-informed conservation strategies.

## 1 Introduction

Global amphibian populations are experiencing an unprecedented decline, with over 40% of species currently threatened with extinction (Stuart et al., 2004). Emerging diseases, habitat loss, invasive species, overexploitation, and pollution are among the primary factors driving this sharp decline (Reid et al., 2019; Roach et al., 2024). The gut microbiota serves as a vital barrier against these pressures, modulating the immune system, aiding nutritional metabolism, and suppressing pathogens (Long et al., 2024a,b; Zhao et al., 2024). Due to amphibians' biphasic life cycle and highly permeable skin, they are extremely sensitive to environmental disturbances associated with human activities, temperature fluctuations, and declining water quality, leading to rapid shifts in their gut microbiota (Dong et al., 2024; Hernández-Gómez and Hua, 2023). Understanding how microbiota respond to environmental disturbances is essential for predicting health risks in amphibians and developing effective conservation strategies (Wang et al., 2025).

The annual global production of straw is ~1 billion tons (Harun et al., 2022). However, the comprehensive utilization of straw remains low, resulting in substantial amounts being discarded or burned directly in fields (Huang et al., 2021). Although field burning rapidly clears straw and reduces pests and plant diseases, it significantly contributes to air pollution, leading to extensive research attention (Dinh et al., 2024; Zhao, 2022). Rice straw burning may also alter soil temperature, pH, and nutrient cycling, thereby affecting agricultural productivity and ecosystem stability (Chen et al., 2022; Singh et al., 2023). However, the pollution pathway of ash entering water bodies through rainfall is indirect and slow, making its environmental impact difficult to detect or quantify (Bodí et al., 2014; Dong et al., 2024). Water pollution is usually caused by multiple factors and requires long-term monitoring, further complicating relevant research efforts (Nie and Liu, 2024; Nong et al., 2024). Regarding the impact of ash on aquatic organisms, most studies have focused on fish ecotoxicology or behavioral responses following ash exposure (Brito et al., 2017; Jaunjal and Gaupale, 2025; Kirsch et al., 2024; Miranda et al., 2025). Studies have demonstrated that acute toxicity of sugarcane ash to fish induces abnormal behaviors such as increased oxygen demand, reduced activity, and decreased feeding rates over short-term exposures (Yofukuji et al., 2021). Tadpoles typically consume algae, detritus, and microorganisms from aquatic surfaces; they also absorb dissolved substances through their permeable skin (Long et al., 2024b; Montaña et al., 2019). Consequently, both life stages will be in prolonged contact with water bodies rich in ash particles and dissolved pollutants, potentially exhibiting markedly different physiological-microbiological responses (Long et al., 2024b; Ranvestel et al., 2004; Roe et al., 2005). Ignoring differences underestimates the risks posed by pollutants (such as rice straw ash) to amphibians, highlighting the urgent need to assess their impact on amphibians and gut microbiota across different developmental stages (Scott-Elliston, 2023).

Wildfire activity around the world is becoming increasingly frequent, the intervals between fires are shortening, and the fire season is lengthening (Jones et al., 2022; Senande-Rivera et al., 2022). In a single wildfire, several tons of highly alkaline ash can be produced per hectare of burned biomass, enriched with soluble ions (e.g., Ca^2+^, K^+^, and Mg^2+^), trace metals (e.g., Cu, Zn, and Pb), and polycyclic aromatic hydrocarbons (PAHs; Long et al., 2024b; Lopez et al., 2023; Sanchez-Garcia et al., 2023). Heavy rainfall following wildfires can wash these ashes into headwater streams, causing an abrupt increase in pH, electrical conductivity, and suspended solids while simultaneously dissolving oxygen (Cooke et al., 2022; Li et al., 2024a; Paul et al., 2022). This physicochemical shock directly kills large invertebrates, inhibits algal growth, and causes sub-lethal stress to fish (Abu-Elala et al., 2025; Anih et al., 2024). Previous studies have shown that various types of ash have a significant impact on the survival and gut and skin microbiota of *Rana dybowskii* (Dong et al., 2024; Tong et al., 2025; Xu et al., 2024). However, the broader impacts on amphibians have been greatly overlooked. The main difference between wildfires and straw burning is that wildfires occur in natural ecosystems, and the ashes contain more toxic substances, such as PAHs and heavy metals, thereby exerting more severe and long-lasting impacts (Long et al., 2024b; Xu et al., 2024). The mineral composition and PAHs content of wildfire ash differ from those of agricultural straw ash, suggesting distinct toxicity profiles (Lopez et al., 2024). Straw burning primarily occurs in agricultural areas, and the ash is usually rich in soluble salts and organic matter containing metal elements (e.g., Al and Ba), which can rapidly induce water eutrophication and adversely affect aquatic organisms (Dong et al., 2024; Long et al., 2024b). However, research on the effects of different types of ash on the gut microbiota of amphibians at various developmental stages remains scarce.

In different developmental stages of amphibians (tadpole vs. adult stages), the composition and function of their gut microbiota may vary significantly (Dong et al., 2024; Tong et al., 2025; Xu et al., 2024). The diversity and complexity of gut microbiota during the tadpole stage are usually lower than in the adult stage, comprising microbial communities adapted to an aquatic, filter-feeding lifestyle (Shen et al., 2022; Zhu et al., 2024a). As amphibians develop, the gut microbiota in the adult stage gradually becomes richer and more diverse, capable of supporting more complex physiological needs, such as digesting a wider variety of foods and effectively defending against foreign pathogens (Zhang et al., 2020). *Rana dybowskii* is widely distributed in Northeast Asia and is highly sensitive to ash and pesticide pollution caused by agricultural activities, and it can lead to significant reductions in population size, developmental conditions, and survival, making it an ideal indicator species for monitoring the ecological effects of agricultural pollution (Dong et al., 2024; Tong et al., 2020a, 2025). Environmental stresses, such as various types of ash, may have different toxicity patterns on the gut microbiota (Dong et al., 2024; Xu et al., 2024). Adult *R. dybowskii* typically hibernate from October each year until March of the following year (Long et al., 2024b). In March and April, the tadpoles of *R. dybowskii* hatch and begin to grow (Petranka and Thomas, 1995). The occurrence of wildfires and straw burning coincides with the activity periods of both tadpoles and adult individuals of *R. dybowskii*, thereby exposing both developmental stages to environmental risks associated with ash pollution (Long et al., 2024b). This study aims to investigate how ash from different sources affects the gut microbiota of *R. dybowskii* at various developmental stages (adult and tadpole) by employing high-throughput sequencing techniques. The following hypotheses are proposed: (1) Wildfire ash has a stronger destructive effect and greater toxicity on the gut microbiota of *R. dybowskii* compared to straw ash; (2) The responses at different developmental stages are specific, with tadpoles predicted to be more sensitive than adults.

## 2 Materials and methods

### 2.1 Experimental materials

Adult *R. dybowskii*, which were used in this study, were collected from Luobei County (47.5789N, 130.3794E), Heilongjiang Province, China. Both female and male individuals were collected from a hibernation pond, where they had begun fasting in November due to decreasing temperatures. The healthy frogs had an average body mass of 20.48 ± 1.16 g. Upon transportation to the laboratory, the frogs were acclimated to laboratory conditions for 7 d before being assigned to their respective experimental groups.

The tadpoles used in this experiment were obtained by breeding adult *R. dybowskii* that were collected in this study from Luobei County (47.5789N, 130.3794E), Heilongjiang Province, China. These adult frogs reproduced after undergoing 5 months of hibernation in a hibernation pond. To simulate the natural hibernation conditions, the frogs were kept in a freezer (Haier BC/BD-307HEM). The tadpoles were raised to Gosner 20 stage (G20) with an average body mass of 0.12 ± 0.01 g in March (Gosner, 1960). The tadpoles were then used in experiments, and on experimental day 28, those reaching G26 were transferred to the laboratory for further analysis.

The rice straw ash used in this study, collected in March 2022 from Zhaoyuan County (45.6990N, 124.2460E), Heilongjiang Province, China, was characterized as black ash. The wildfire ash in 2019 was collected near Jiamusi city (46.7332N, 130.1301E) in Heilongjiang Province, China. The wildfire took place in a mountainous forest characterized by Chinese fir and deciduous larch flora. Ecotoxicological tests were conducted using gray-white ash collected from wildfires that ranged from moderate to high severity (Coelho et al., 2022; Xu et al., 2024).

Immediately after the fire, a 50 × 50 cm area was marked for ash retrieval at each site, used a spoon and brush to transfer ash, which was then sieved through a 2 mm mesh to avoid soil contamination (Dong et al., 2024). Collected samples were placed in plastic bags, homogenized in the laboratory, and stored at −18 °C in darkness to suppress biological activity prior to subsequent analysis and ecotoxicological evaluation (Xu et al., 2024).

### 2.2 Experimental grouping, concentration selection, and animal feeding

The experiment assessed the effects of aqueous extracts of ash (AEAs) on survival and gut microbiota of tadpoles and adult frogs using six groups: tadpole control (TCG, 0 g·L^−1^), tadpole straw ash (TSG, 6 g·L^−1^), tadpole wildfire ash (TWG, 6 g·L^−1^) for 28 d (three replicates, 35 tadpoles each); frog control (FCG, 0 g·L^−1^), frog straw ash (FSG, 10 g·L^−1^), and frog wildfire ash (FWG, 10 g·L^−1^) for 30 d (three replicates, 10 frogs each).

According to previous studies on ash, Australian wildfire ash exhibits high toxicity to *Daphnia magna*, with a complete mortality observed within 24 h at a concentration of 25 g L^−1^, while lower concentrations (6.25 and 12.5 g L^−1^) result in mortality rates of about 10 and 75%, respectively (Harper et al., 2019). We conducted acute toxicity tests in which adult *R. dybowskii* and tadpoles were exposed to concentrations of 5, 10, and 15 g L^−1^. The results showed that after 48 h of exposure at 15 g L^−1^, mortality occurred in both tadpoles and adults. At a concentration of 10 g L^−1^, mortality was observed in tadpoles within 48 h of exposure, while no deaths were observed in adults. Subsequently, tadpoles were exposed to 6 and 8 g L^−1^ for 48 h. Mortality was observed at 8 g L^−1^, while no deaths occurred at 6 g L^−1^. Based on these findings, we selected 10 and 6 g L^−1^ as the high-concentration exposure groups for adults and tadpoles, respectively, in subsequent experiments to further assess the effects of extreme ash concentrations on different developmental stages of *R. dybowskii*.

AEAs were prepared under strictly controlled conditions for use in ecotoxicity assays (Santos et al., 2023a). AEA solutions containing straw or wildfire ash were prepared at a concentration of 10 g L^−1^ by dissolving 400 g of ash in 40 L of artificial water (Dawson and Bantle, 1987; Santos et al., 2023a). Following the protocols described in our previous studies, we prepared AEA solutions at a concentration of 10 g·L^2+^ (Dong et al., 2024; Long et al., 2024b; Xu et al., 2024). To reduce the concentration, each liter of the original solution was diluted by adding 0.67 L of water, resulting in a final concentration of 6 g L^−1^ for subsequent tadpole exposure experiments (Long et al., 2024b).

The overwintering conditions for adult *R. dybowskii* were established in a controlled laboratory setting, with hibernation barrels maintained at 1.1 ± 0.2°C. The photoperiod was set to a 12 h day-night cycle. The barrels for hibernation consisted of a white polyethylene cylinder (25 L). *Rana dybowskii* entered a fasting state during hibernation. Frogs were carefully introduced into these barrels for the immersion phase. Mortality was monitored and recorded daily. AEAs solutions were refreshed every 5 d throughout the 30 d study period. There were 10 frogs assigned to each group.

Each 40 L collection was placed into individual 50 L oxygenated polyethylene barrels. To ensure adequate aeration and oxygenation, each experimental group was continuously supplied with oxygen via an air pump connected to an air stone. After mixing, each group reached a stable pH range of 7.6–7.9. Tadpoles at Gosner stage 20 (G20; 0.12 ± 0.01 g) were immersed in the designated AEA solutions, which were completely renewed every 5 d over a 28-day experimental period, until individuals reached G26. Conditions included water at 15.9 ± 1.8°C, daily feeding (fish food tablets and rabbit chow), 12 h light/dark cycles, daily survival checks, and removal of deceased tadpoles and waste to maintain water quality (Long et al., 2024b). Each experimental group contained 35 tadpoles.

### 2.3 Sample selection

On day 30, frog specimens were rapidly euthanized at the Jiamusi University laboratory for gut microbiota analysis, with nine individuals randomly selected per group. Frogs were euthanized by sedation with an ether-alcohol-soaked cotton ball in a glass desiccator, followed by mechanical injury via insertion of a metal rod through the foramen magnum into the brain and spinal cord. On day 28, tadpole samples (G26) were euthanized in Petri dishes using tricaine methanesulfonate (MS-222) and alcohol anesthesia, with three tadpoles sampled per container for each group's total of nine gut samples. After confirming death, gastrointestinal tracts (excluding tails and toes in tadpoles and stomachs in frogs) were immediately excised, washed with ultrapure water (tadpoles only), and the intestinal contents carefully transferred to sterile 5 mL containers using aseptic instruments. Samples were rapidly frozen at −80°C until further analysis.

### 2.4 DNA extraction and PCR amplification

A FastDNA^®^ spin kit for soil (MP Biomedical, US) was used to extract microbial DNA from gut microbiota after the material had been homogenized, by the manufacturer's instructions. The A260/A280 ratio and DNA concentration were assessed using a NanoDrop 2000 spectrophotometer (Thermo Scientific, US), while DNA quality was analyzed by 1% agarose gel electrophoresis. Using primers 338F (5′-ACTCCTACGGGAGGCAGCAG-3′) and 806R (5′-GGACTACHVGGGTWTCTAAT-3′), the 16S rRNA genes of bacteria found in the V3–V4 regions were amplified. The PCR procedure was repeated 27 times, starting with a 3 min denaturation at 95°C, followed by 0.5 min denaturation at 95°C, 0.5 min annealing at 55°C, and 0.75 min extension at 72°C. The last extension was performed for 10 min at 72°C. The PCR mixture, consisting of 4 μL of 5 × FastPfu Buffer, 0.4 μL of FastPfu polymerase, 2 μL of 2.5 mM dNTPs, 10 ng of template DNA, and 0.8 μL of each primer (5 μM), was added to sterilized double-distilled water (ddH_2_O) in a total volume of 20 μL. The AxyPrep DNA gel extraction reagent from Axygen Biosciences, a US-based company, was used to extract the PCR products from a 2% agarose gel and purify them. The QuantiFluor™-ST test instrument from Promega, a US-based company, was employed to quantify DNA.

### 2.5 Illumina MiSeq sequencing

Following the completion of amplicon level standardization, the collected samples were submitted to library quality control, quantification, and paired-end sequencing (2 × 300) utilizing a MiSeq platform that was manufactured by Illumina and came from a company located in the United States. Sequencing was performed at Majorbio Bio-Pharm Technology Co., Ltd. (Shanghai, China). Following are the accession numbers that have been assigned to the microbiota sequences that have been uploaded to the SRA database of the National Center for Biotechnology Information's (NCBI), including PRJNA1050069 (TWG6 group), PRJNA1050064 (TCG0 group), PRJNA1050067 (TSG6 group), PRJNA1037587 (FCG0 group), PRJNA1040936 (FTG10 group), and PRJNA1037759 (FSG10 group).

### 2.6 Processing of sequencing data

Raw fastq files were demultiplexed, quality filtered using Trimmomatic, and then combined using FLASH based on the following parameters. Initially, we truncated 300-base pairs (bp) reads with an average quality score below 20 across a 50-bp sliding window to guarantee that only reads that were 50 bp or longer were retained for analysis. Secondly, sequences with overlaps more than 10 bp were built from overlapping sequences, whereas unassembled reads were excluded. Third, we eliminated sequences with erroneous barcodes, two nucleotide mismatches in the primer, and ambiguous characters. Following a 97% similarity criterion, operational taxonomic units (OTUs) were grouped using UPARSE 7.1; chimeric sequences were eliminated with UCHIME. The Silva (SSU138) 16S rRNA database was utilized to taxonomically classify each and every 16S rRNA gene sequence, with a 70% confidence level.

### 2.7 Ecological and statistical analysis

Survival in this study was analyzed using one-way analysis of variance (ANOVA), and significant differences were identified through Tukey's HSD test and Benjamini-Hochberg FDR correction in R (version 3.3.1). Statistical significance was defined as a *P* < 0.05. Tukey HSD enabled the assessment of disparities among many groups in survival analysis. The mothur program (version v.1.30.2, https://mothur.org/wiki/calculators/) was utilized to generate rarefaction curves and evaluate alpha diversity markers for gut microbiota, including the abundance-based coverage estimators (ACE), the Chao1 estimator (Chao), the Shannon index (Shannon), and the observed richness (Sobs; Hadizadeh et al., 2017). Multiple testing approaches, including the FDR-corrected Kruskal-Wallis *H*-test, were used to analyze the data. Only *P* values < 0.05 were shown. The gut microbiota of the frog and tadpole were considered dominant if they were found in 90% of the samples and contributed more than 0.1% of the sequencing reads. Venn diagrams were used to visualize the shared and unique OTUs among multiple samples at the 97% OTU similarity level. Statistics and plotting were performed using R (version 3.3.1).

The beta diversity distance matrices were computed with the help of Qiime (https://qiime.org), and the non-metric multidimensional scaling (NMDS) analysis and mapping were carried out using the vegan package (version 2.4.3) in R (version 3.3.1; Knights et al., 2011). The influence of environmental factors on community clustering and group dispersion was investigated using Bray-Curtis dissimilarities and weighted UniFrac distances obtained from an OTU-level table, Analysis of Similarity (ANOSIM), and Multivariate Non-parametric Analysis of Variance (Adonis, 999 permutations).

Based on the data in the tax_summary_a folder, bar plots generated using R (version 3.3.1) visually displayed the taxonomic composition and relative abundance of dominant species at various taxonomic levels across different groups. The relative abundance variations across many groups were assessed using the multiple test correction (Benjamini-Hochberg FDR) and Kruskal-Wallis *H*-test. Results were shown only when *P* < 0.05. A ternary plot that was created with the “ggtern” and “ggplot2” packages demonstrated the degree to which these groupings were connected to one another, and the species that were dominant (>0.5% in at least one sample) were distributed (Xie et al., 2023).

The linear discriminant analysis effect size (LEfSe, LDA > 4) was employed to identify unique phyla and genera, combining biological relevance and statistical significance (Lian et al., 2019). BugBase, a software tool for microbiota analysis, detected and predicted prominent phenotypic features present in microbial samples (Lucas et al., 2018). BugBase used pre-calculated files to evaluate microbiota features by standardizing OTUs about predicted 16S rRNA gene copy counts. Differences in relative abundance between the control, straw ash, and wildfire ash groups were assessed using the Kruskal-Wallis *H*-test. Only data with *P* < 0.05 were shown.

## 3 Results

### 3.1 Survival

**Figure 1:**
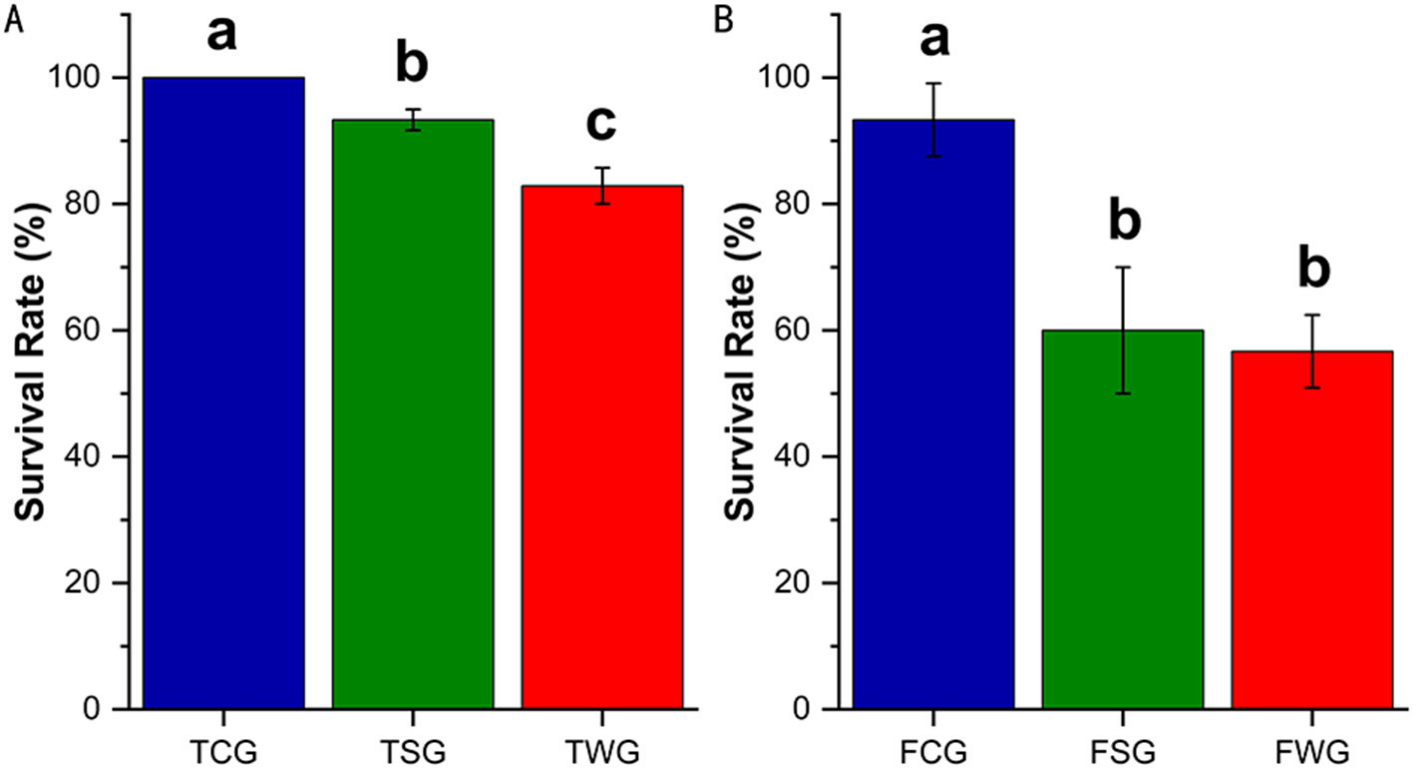
Survival rate of *R. dybowskii* adults and tadpoles exposed to various types of ashes. The level of significance compared to the control was represented by “a,” “b,” and “c.” One-way ANOVA followed by Tukey's HSD test and Benjamini-Hochberg FDR correction was used to analyze the statistical difference between the control and the experiments. **(A)** Adults stage survival rate of rice straw ash and wildfire ash (30 d). **(B)** Survival rate of the tadpole stage of rice straw ash and wildfire ash (28 d).

One-way ANOVA demonstrated significant survival differences among TCG, TSG, and TWG (*P* = 0.001), with each pairwise comparison remaining significant (adjusted *P* = 0.001; Figure 1A). Analysis of variance among the FCG, FSG, and FWG groups revealed a significant difference (*F* = 22.20, *P* = 0.002), with a significant *post-hoc* result between FCG and FSG, FCG and FWG (adjusted *P* = 0.001), but not between FSG and FWG (adjusted *P* = 0.900; Figure 1B). Compared to rice straw ash, wildfire ash resulted in lower survival of *R. dybowskii* at different developmental stages (Figures 1A, B).

### 3.2 Alpha diversity and shared microbiota

**Figure 2:**
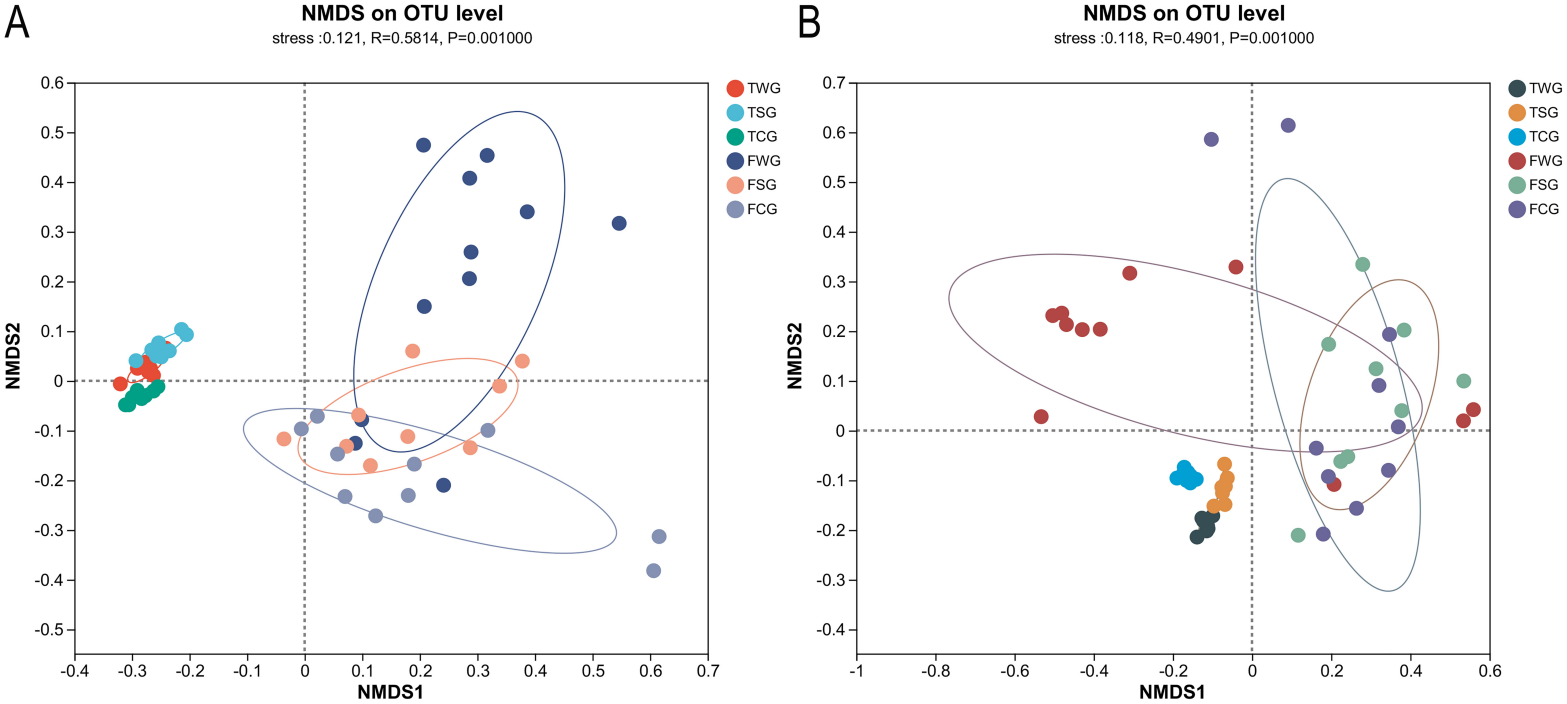
Examining the influence of straw ash and wildfire ash on frogs and tadpoles' microbiota beta diversity utilizing Non-metric multidimensional scaling (NMDS) analysis. Each data dot signifies a specific sample procured from the gut, and each color represents a distinct group. Showing the 95% confidence ellipses for samples taken on six groups. **(A)** The Bray-Curtis dissimilarity matrix was used to perform NMDS analysis comparing adults and tadpoles in the control, rice straw ash, and wildfire ash groups. **(B)** NMDS based on the weighted-UniFrac distance matrix of adults and tadpole stages at control, rice straw ash, and wildfire ash groups.

Sequencing depth was efficiently represented using rarefaction and Shannon curves (Supplementary Figures 1A, B). The Rarefaction curve leveled off, indicating adequate sequencing depth for the study (Supplementary Figure 1B). The microbial alpha diversity (ACE, Chao, Shannon, and Sobs indices) differed significantly among the TCG, TSG, and TWG groups (Kruskal-Wallis *H*-test and Tukey-Kramer *post-hoc* analysis, *P* < 0.05; Supplementary Figure 2A). There were no variations in the TCG and TWG groups' ACE and Shannon indices (Wilcoxon test and Tukey-Kramer *post-hoc* test, *P* > 0.05; Supplementary Figure 2A). Comparing ACE indices between TSG and TWG groups showed no statistically significant variation (Wilcoxon test and Tukey-Kramer *post-hoc* test, *P* > 0.05; Figure 2A). The TSG group exhibited higher ACE, Chao, Shannon, and Sobs indices compared to the TCG and TWG groups (*P* > 0.05; Supplementary Figure 2A). The microbial alpha diversity (ACE, Chao, Shannon, and Sobs indices) did not significantly differ across the FCG, FSG, and FWG groups (Kruskal-Wallis *H*-test, *Post-hoc* test: Tukey-Kramer, *P* > 0.05; Supplementary Figure 2B).

A shared set of 155 OTUs was identified that was shared across TCG, TSG, and TWG groups (Supplementary Figure 3A). TCG group displayed 101 unique OTUs, TSG group 231, and TWG group 124 (Supplementary Figure 3A). With a shared subset of 276 OTUs across FCG, FSG, and FWG groups (Supplementary Figure 3B). FCG group had 67 unique OTUs, FSG group 91, and FWG group 58 (Supplementary Figure 3B).

### 3.3 Beta diversity

The gut microbial beta diversity of the TCG, TSG, and TWG groups differed significantly, according to the Bray-Curtis dissimilarity matrix (Adonis: *R*^2^ = 0.788, *P* = 0.001; ANOSIM: statistic = 0.988, *P* = 0.001; Table 1, Figure 2A and Supplementary Figure 4A) and weighted UniFrac distances (Adonis: *R*^2^ = 0.793, *P* = 0.001; ANOSIM: statistic = 0.947, *P* = 0.001; Table 1, Figure 2B and Supplementary Figure 4B). The TCG, TSG, and TWG samples were tightly spaced and had clear clustering (Figure 2 and Supplementary Figure 4). Significant Bray-Curtis and weighted UniFrac distance differences were observed between TCG and TSG, TCG and TWG, and TSG and TWG groups (Bray-Curtis: ANOSIM, *P* < 0.05; weighted UniFrac: ANOSIM, *P* < 0.05; Table 1).

**Table 1:** Using pairwise comparisons, variations in the gut microbiota among groups were determined.

	**Bray-Curtis**				**Weighted UniFrac**			
	**ANOSIM**		**Adonis**		**ANOSIM**		**Adonis**	
	**Statistic**	***P***	*R* ^2^	***P***	**Statistic**	***P***	*R* ^2^	***P***
TCG vs. TSG	1.000	0.001	0.774	0.001	0.994	0.001	0.789	0.001
TCG vs. TWG	1.000	0.001	0.806	0.001	1.000	0.001	0.828	0.001
TSG vs. TWG	0.939	0.001	0.600	0.001	0.739	0.001	0.463	0.001
FCG vs. FSG	0.031	0.259	0.075	0.139	0.037	0.234	0.081	0.159
FCG vs. FWG	0.385	0.002	0.150	0.003	0.375	0.002	0.223	0.005
FSG vs. FWG	0.278	0.007	0.154	0.007	0.373	0.005	0.250	0.005

C stands for control group, F stands for frog, G stands for gut, S stands for straw ash, T stands for tadpole, and W stands for wildfire ash.

Significant differences in gut microbial beta diversity among the FCG, FSG, and FWG groups were observed, as indicated by the Bray-Curtis dissimilarity matrix (Adonis: *R*^2^ = 0.168, *P* = 0.002; ANOSIM, statistic = 0.246, *P* = 0.001; Figure 2A and Supplementary Figure 4C) and weighted UniFrac distances (Adonis: *R*^2^ = 0.224, *P* = 0.002; ANOSIM, statistic = 0.264, *P* = 0.001; Figure 2B and Supplementary Figure 4D). NMDS analysis demonstrated that the three groups were generally close in ordination space, FCG and FWG had more dispersed distributions, whereas FSG samples were more tightly clustered (Figure 2 and Supplementary Figure 4). Significant differences among FCG vs. FWG groups and FSG vs. FWG groups (Bray-Curtis: ANOSIM, *P* < 0.05; weighted UniFrac: ANOSIM, *P* < 0.05; Table 1), but no FCG or FSG groups (Bray-Curtis: ANOSIM, *P* > 0.05; weighted UniFrac: ANOSIM, *P* > 0.05; Table 1).

### 3.4 Composition and differences in the gut microbiota

**Figure 3:**
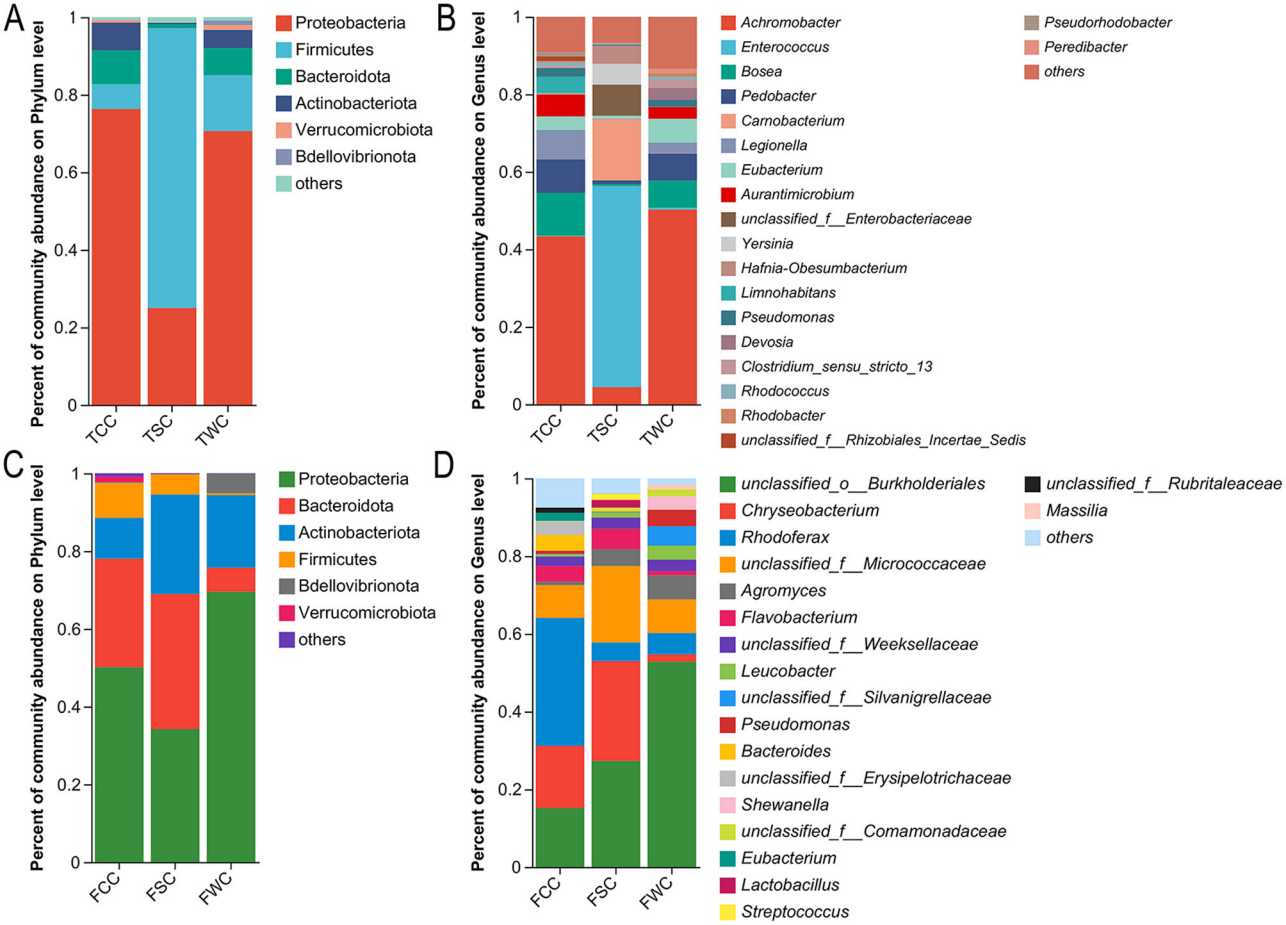
Relative abundances of gut microbiota compositions in control, straw ash, and wildfire ash adults and tadpoles. Show only those phyla and genera taxa with relative abundances exceeding 1% in a minimum of one sample. Relative abundances of the dominant phyla **(A,C)** and genera **(B,D)** of adults and tadpoles' gut microbiota. The x-axis represents the sample names, and the y-axis represents the proportion of species in each sample. Different colored bars represent different species, and the length of the bars indicates the proportion of each species.

Group TCG, TSG, and TWG gut microbiota composition primarily comprised Firmicutes (51.77, 87.87, and 88.59%), Proteobacteria (25.57, 5.47, and 8.32%), Actinobacteriota (21.55, 3.95, and 1.88%), and Fusobacteriota (0.15, 2.13, and 0.35%; Figure 3A and Supplementary Figure 5A). Significant differences in 10 of 16 phyla were noted among TCG, TSG, and TWG (Kruskal-Wallis *H*-test and multiple test correction; Benjamini-Hochberg FDR, adjusted *P* < 0.05; Figure 3A and Supplementary Figure 5A).

TCG group's microbiota composition was led by *Eubacterium* (42.17%), *Aurantimicrobium* (21.10%), *Legionella* (17.24%), *[Anaerorhabdus]_furcosa_group* (3.73%), and *unclassified_f__Rhizobiales_Incertae_Sedis* (3.72%; Figure 3B and Supplementary Figures 5B, 6). TSG group's microbiota composition main genera were *Eubacterium* (38.75%), *Alkaliphilus* (14.31%), *Anaerocolumna* (5.65%), *Enterococcus* (3.55%), and *Exiguobacterium* (3.35%; Figure 3B and Supplementary Figures 5B, 6). *Eubacterium, Exiguobacterium, Anaerocolumna, Bosea*, and *Enterococcus* dominated the TWG group microbiota composition, accounting for 53.65, 15.34, 6.06, 5.67, and 3.02% of relative abundance (Figure 3B and Supplementary Figures 5B, 6). Significant differences were observed in 135 of 307 gut microbiota genera (Kruskal-Wallis *H*-test and Benjamini-Hochberg FDR multiple test correction, *P* < 0.05; Figure 3B and Supplementary Figures 5B, 6).

In the FCG, FSG, and FWG groups microbiota composition, Firmicutes (46.94, 55.53, and 17.59%), Proteobacteria (22.87, 11.31, and 62.23%), Bacteroidota (16.02, 26.98, and 11.65%), and Actinobacteriota (7.76, 4.19, and 3.24%) were the dominant phyla in the gut microbiota (Figure 3C and Supplementary Figure 5A). Only Fusobacteriota differed significantly among TCG, TSG, and TWG groups out of 17 phyla (Kruskal-Wallis *H*-test and multiple test correction; Benjamini-Hochberg FDR, adjusted *P* < 0.05; Figure 3C and Supplementary Figure 5A).

FCG group's microbiota composition was dominated by *Rhodoferax* (15.76%), *unclassified_f__Erysipelotrichaceae* (7.05%), *Gordonibacter* (5.90%), *Bacteroides* (5.02%), and *Eubacterium* (4.99%; Figure 3D, Supplementary Figures 5B, 6). FSG group's microbiota composition dominant genera included *Bacteroides* (12.61%), *unclassified_f__Erysipelotrichaceae* (10.76%), *unclassified_f__Weeksellaceae* (10.47%), *unclassified_f__Lachnospiraceae* (7.42%), and *Eubacterium* (5.34%; Figure 3D and Supplementary Figures 5B, 6). The FWG group's microbiota composition, with the main genera being *Pseudomonas* (35.00%), *Hafnia-Obesumbacterium* (13.41%), *Shewanella* (8.90%), *Bacteroides* (5.61%), and *Eubacterium* (1.81%; Figure 3D and Supplementary Figures 5B, 6). Substantial variation was noted in one (*unclassified_c__Gammaproteobacteria*) of 263 gut microbiota genera (Kruskal-Wallis *H*-test and Benjamini-Hochberg FDR multiple test correction, *P* < 0.05; Figure 3D and Supplementary Figures 5B, 6).

### 3.5 Dominant bacterial taxa composition and abundance across groups

*Eubacterium* (Firmicutes) averaged 44.90% in TCG, TSG, and TWG, with abundances of 31.30, 28.80, and 39.90%, respectively (Supplementary Figure 7A). *Pseudomonas* (Proteobacteria) had 12.10% relative abundance, with 0.20, 3.10, and 96.70% in FCG, FSG, and FWG (Supplementary Figure 7B). *Clostridium_sensu_stricto_13* (Firmicutes) had 0.90% overall abundance, with 4.20% in FCG, 94.00% in FSG, and 1.80% in FWG (Supplementary Figure 7B). *Unclassified_f__Ruminococcaceae* (Verrucomicrobiota) showed 0.90% abundance: 99.90% in FCG, 0.10% in FWG, absent in FSG (Supplementary Figure 7B).

### 3.6 Diversification of gut microbiota in frogs and tadpoles

**Figure 4:**
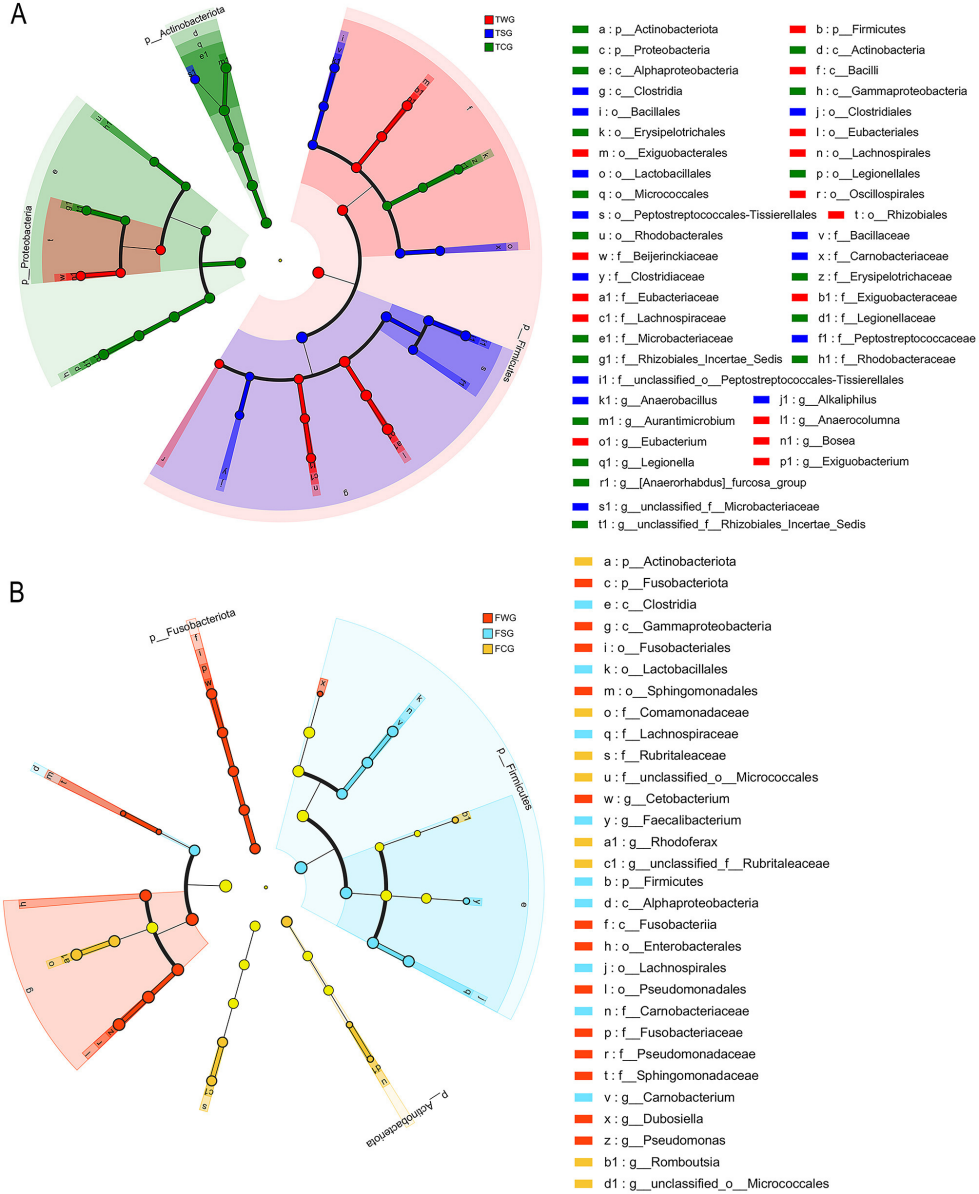
Cladogram of linear discriminant analysis effect size (LEfSe) results (from phylum to genus level) according to the different groups. Post-treatment color differentiation was classified into multiple distinct groups. The FCG/TCG group represents control samples, FSG/TSG group indicates straw ash samples, FWG/TWG group represents wildfire ash samples, and F indicates frog stage **(B)** and T indicates tadpole stage **(A)**. The abundance of each group was shown by the diameter of the corresponding circle. A versatile multiclass analysis displays at least one class difference. Inner to outer circles represent taxonomic classifications from domain to genus. Depictions of phylum, class, order, family, and genus labels were present. Taxa exhibiting an LDA > 4 were illustrated.

LEfSe analysis showed Actinobacteriota and Proteobacteria enrichment in TCG group (LDA > 4, *P* < 0.05; Figure 4A). LEfSe analysis at genus level revealed significant TCG group enrichment of *Aurantimicrobium, Legionella, unclassified_f__Rhizobiales_Incertae_Sedis*, and *[Anaerorhabdus]_furcosa_group* (LDA > 4, *P* < 0.05; Figure 4A). LEfSe analysis showed no significant phylum-level differences in TSG group (LDA > 4, *P* > 0.05; Figure 4A). Genus-level LEfSe analysis revealed significant enrichment of *Alkaliphilus, Anaerobacillus*, and *unclassified_f__Microbacteriaceae* in TSC group (LDA > 4, *P* < 0.05; Figure 4A). The LEfSe analysis indicated a significant increase in the abundance of Firmicutes within the TWG group (LDA > 4, *P* < 0.05; Figure 4A). At the genus level, the LEfSe analysis revealed a significant enrichment of *Anaerocolumna, Bosea, Exiguobacterium*, and *Eubacterium* within the TWG group (LDA > 4, *P* < 0.05; Figure 4A).

LEfSe analysis revealed significant enrichment of Actinobacteriota and Proteobacteria in FCG group (LDA > 4, *P* < 0.05; Figure 4B). LEfSe analysis identified significant enrichment of *Rhodoferax, Romboutsia, unclassified_o__Micrococcales*, and *unclassified_f__Rubritaleaceae* in the FCG group (LDA > 4, *P* < 0.05; Figure 4B). LEfSe analysis results indicated a significant rise in Firmicutes abundance in the FSG group (LDA > 4, *P* < 0.05; Figure 4B). In the FSG group, LEfSe analysis revealed a notable predominance of genera such as *Carnobacterium* and *Faecalibacterium* (LDA > 4, *P* < 0.05; Figure 4B). LEfSe analysis showed a significant difference in Fusobacteriota abundance in the FWG group (LDA > 4, *P* < 0.05; Figure 4B). In the FWG group, LEfSe analysis revealed notable genus-level differences, including *Cetobacterium, Dubosiella*, and *Pseudomonas* (LDA > 4, *P* < 0.05; Figure 4B).

### 3.7 Estimating bacterial phenotypes with the BugBase method

**Figure 5:**
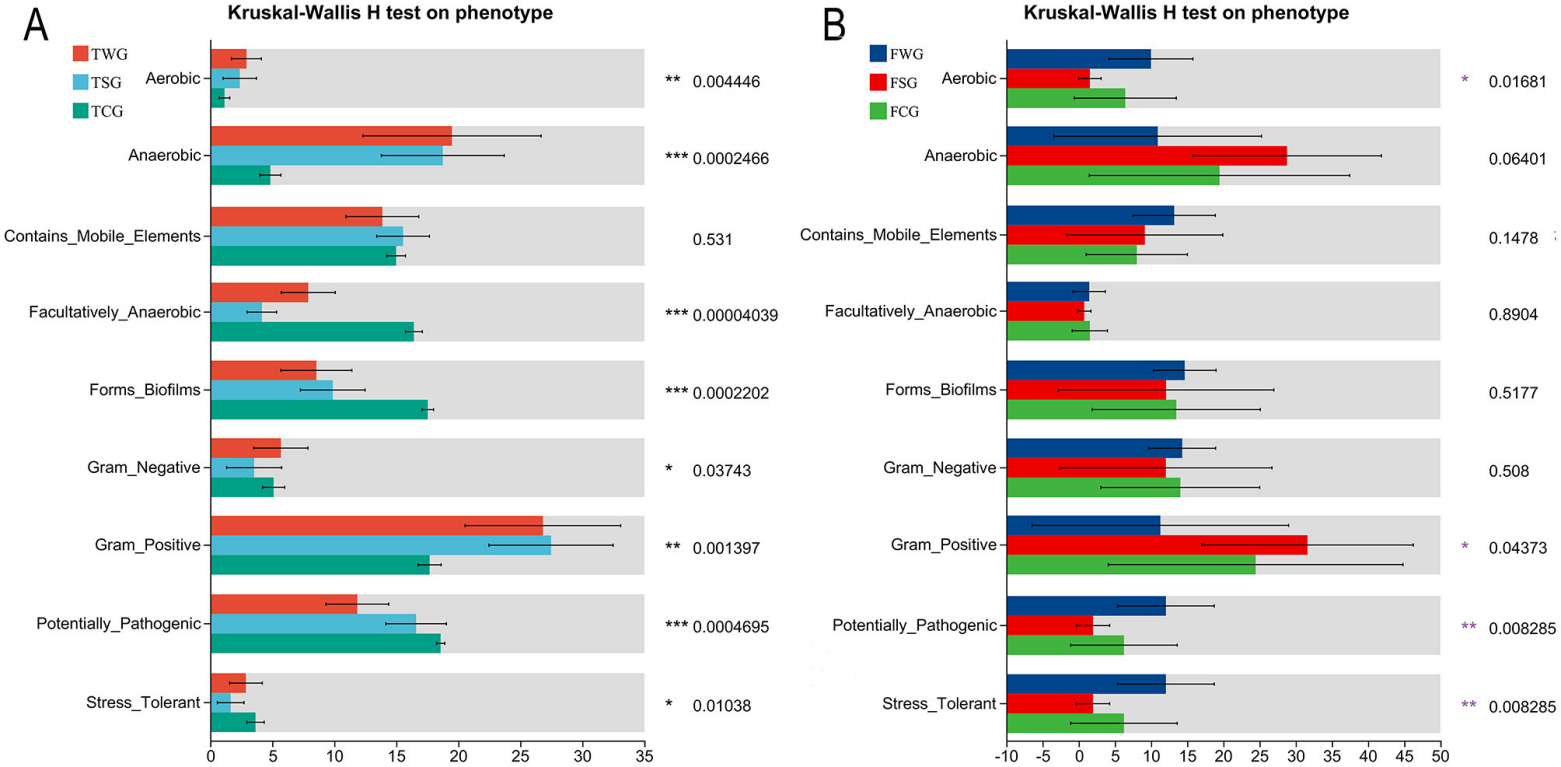
Phenotype prediction of bacteria by BugBase analysis. Assessing the influence of straw ash and wildfire ash on adult **(B)** and tadpole **(A)** gut microbiota phenotypes, bacterial characteristics were examined and projected using the BugBase approach. The Kruskal-Wallis *H*-test was used to analyze the statistical difference between the control and the experimental groups. *P* is the significant difference value: *P < 0.05, **P < 0.01, ***P < 0.001.

BugBase analysis of the TCG, TSG, and TWG groups revealed eight phenotypes with significant variations, including Biofilm-forming phenotype and Potentially-pathogenic. In the TCG group, four phenotypes, namely Facultatively-anaerobic, Forms-biofilms, Potentially-pathogenic, and Stress-tolerant, were significantly higher than those in the other two groups. TSG group showed significantly higher Gram-positive phenotypes than TCG and TWG groups. The Aerobic, Anaerobic, and Gram-negative phenotypes of the TWG group were considerably greater than those of the TCG and TSG groups (Kruskal-Wallis *H*-test, *P* < 0.05, Figure 5A).

Analysis of FCG, FSG, and FWG groups using the BugBase algorithm revealed four distinct phenotypes include Aerobic, Gram-positive, Potentially-pathogenic, and Stress-tolerant. The FWG group showed significantly higher Aerobic, Potentially-pathogenic, and Stress-tolerant phenotypes than FCG and FSG groups. The FSG group showed a higher prevalence of gram-positive bacteria than FCG and FWG groups (Kruskal-Wallis *H*-test, *P* < 0.05, Figure 5B).

## 4 Discussion

### 4.1 Survival

This study found that the TWG group had a lower tadpole survival than the TCG and TSG groups. The adult survival was similar between the FSG and FWG groups but lower than that of the FCG group. This indicated that tadpoles were more sensitive to environmental stress and that wildfire ash exhibited higher toxicity compared to other types of ash. This may be because, during the tadpole stage, individuals had weaker adaptive abilities. This allowed ash to easily enter through food, directly impacting gut microbiota, causing a microecological imbalance, and exacerbating toxic reactions, ultimately leading to tadpole mortality (Santos et al., 2023a,b; Shen et al., 2022; Tong et al., 2025). Since the adults were fasting, ash was primarily absorbed through the skin, potentially affecting the gut microbiota via the skin-gut axis (Coelho et al., 2022; Mani et al., 2021; Zhu et al., 2024b). Due to limited overall exposure, adults exhibited weaker stress responses to different types of ash, showing higher tolerance and a reduced impact of treatment on survival (Long et al., 2024b). The higher toxicity of wildfire ash is primarily attributed to its complex toxic organic compounds, abundant soluble salts, strong oxidizing substances, high particle activity, and significant disruption to microbial community structure and function (Sanchez-Garcia et al., 2023; Tong et al., 2025). Wildfires produce high temperatures and complex fuel sources that generate various toxic organic substances, such as PAHs and dioxins, which are highly toxic to amphibians, although some heavy metals like Hg and PAHs may be more concentrated in rice straw ash (Dong et al., 2024; Long et al., 2024b; Xu et al., 2024). Wildfire ash contains high levels of highly soluble inorganic salts and oxidizing substances that can impair gut and skin barrier functions, leading to ion imbalance and tissue damage (Romero-Matos et al., 2023; Villarruel et al., 2024). Wildfire ash typically consists of fine particulate matter that is easily suspended and absorbed, enhancing its toxic effects (Tong et al., 2025). Although certain heavy metals such as Hg (194,389 vs. 0.163 μg/L) and PAHs like fluoranthene (192 vs. 123 μg/L) were found in higher concentrations in rice straw ash, wildfire ash may contain undetected synergistic components or greater combined toxicity, resulting in higher overall hazard (Dong et al., 2024; Long et al., 2024b; Xu et al., 2024). Wildfire ash disrupted the structure and function of the gut microbiota, weakened the immune barrier, and reduced adaptive capacity, thereby increasing susceptibility to injury (Long et al., 2024b).

Our study found that the survival of the FCG group was lower (93.33%), whereas that of the TCG group was 100%. In breeding farms, the overwintering survival of *R. dybowskii* typically exceeds 95% over a hibernation period of more than 5 months, with deaths usually occurring at the end of winter. Our experiment lasted only 1 month, and the survival of the FCG group was 93.33%. This may have been due to the overwintering environment in this study failing to meet the physiological needs of *R. dybowskii*, because the lack of suitable hibernation shelter (e.g., rocks) may have hindered stable dormancy (Tong et al., 2023a). However, adding shelters such as rocks to the experimental system could introduce exogenous microorganisms or impurities, reducing environmental controllability and the repeatability of results. Disturbances such as light, noise, and frequent handling in the laboratory may disrupt normal hibernation rhythms, induce stress responses, and affect survival (Ferrie et al., 2014; Tennessen et al., 2014). The low survival may also be related to factors such as exposure conditions, ash composition, and the physiological and microecological sensitivities of various developmental stages (Santos et al., 2023b; Strong et al., 2017). In the experiment, the ash exposure concentration for adult frogs (10 g L^−1^) was higher than that for tadpoles (6 g L^−1^), potentially causing greater toxicity to physiological functions such as skin respiration and liver metabolism, which could have increased mortality (Jayawardena et al., 2017). The experimental rearing density was high (10 individuals per 20 L vs. 35 individuals per 50 L). Adult frogs had a higher body mass (20.48 g), whereas tadpoles' body mass was only 0.12 g. If the water change frequency was insufficient for *R. dybowskii*, the buildup of toxic metabolic by-products could have led to lower survival in the FCG group (Tattersall and Ultsch, 2008). High density may increase environmental stress on *R. dybowskii*, resulting in stress responses and immunosuppression in adult frogs, which may lead to lower survival (Johansson et al., 2010). Future experimental designs should thoroughly consider the ecological and physiological needs of *R. dybowskii* at different developmental stages to optimize conditions and enhance the ecological relevance and interpretability of the data (Brooks and Kindsvater, 2022; Long et al., 2024b).

### 4.2 Beta diversity

This is the first study to compare the effects of rice straw ash and wildfire ash and to show that both can significantly alter the structure of the gut microbiota in tadpoles and adult frogs. In adult frogs, only the FWG group differed significantly from both the FCG and FSG groups, whereas the FCG and FSG groups did not differ from each other. Our results suggest that wildfire ash is more toxic, and tadpoles are more sensitive to exposure to ash (Santos et al., 2023a,b). This further supports our hypothesis. Ash exposure at the tadpole stage could significantly alter the gut microbiota's structure in the short term (Tong et al., 2025). The community distributions of the TCG, TSG, and TWG groups were clearly separated, indicating that the gut micro-ecosystem of tadpoles during early developmental stages may be highly sensitive to ash disturbance and that environmental pollutants can rapidly reshape the gut microbiota (Tong et al., 2025; Xu et al., 2024). At the adult stage, wildfire ash significantly disturbed the gut micro-ecosystem, while rice straw ash showed no difference, indicating that adult frogs may exhibit greater microecological stability or a higher recovery capacity from rice straw ash exposure (Dong et al., 2024; Xu et al., 2024). These results suggest that wildfire ash overcomes this “barrier effect” and significantly alters the structure of the gut microbiota (Tong et al., 2025). NMDS results indicated that samples from the FCG, FSG, and FWG groups were more dispersed, suggesting greater disturbance in the gut micro-ecosystem and higher community heterogeneity (Wu et al., 2025). This study used a single-control experimental design, yet in natural environments, ashes are often co-exposed or combined with other pollutants (Li et al., 2024b). Meanwhile, natural temperature fluctuations and climate warming may further intensify the ecotoxicity of ash (Jeong et al., 2024; Khan et al., 2006). Therefore, future research should focus on the ecological effects of compound pollution, such as rice straw ash combined with pesticides or wildfire ash combined with flame retardants (Burgos-Aceves et al., 2024; Singh et al., 2021).

### 4.3 Variations in the composition of microbiota

In this study, LEfSe analysis showed that the gut microbiota of the TCG, TSG, and TWG groups exhibited significant enrichment at the genus level. This suggested that the addition of exogenous ash disrupted the gut microbiota balance and led to the enrichment of specific dominant genera (Dong et al., 2024; Xu et al., 2024). In the TCG group, *Aurantimicrobium* and *Legionella* were significantly enriched. *Aurantimicrobium*, a dominant genus in healthy tadpole guts, may be crucial for organic matter decomposition, carbon cycling, and the maintenance of gut microecological stability (Han et al., 2025). *Legionella*, a key environmental indicator bacterium, was highly abundant in this group, reflecting the cleanliness of the experimental water and the balance of its native microbiota, which were typical features of gut microbiota during healthy developmental stages (Wang et al., 2024). In the TSG group, *Alkaliphilus* and *Anaerobacillus* were significantly enriched. *Alkaliphilus* can adapt to high pH environments and degrade organic pollutants in ash. Its enrichment in the tadpole gut may contribute to metabolic homeostasis in response to environmental changes caused by alkaline minerals and organic matter released from rice straw ash (Luo et al., 2022). The enrichment of *Anaerobacillus* may indicate changes in the gut's redox state caused by straw ash, promoting the proliferation of anaerobic bacteria that primarily perform anaerobic decomposition and nitrate reduction (Sumithra et al., 2024; Tiso and Schechter, 2015). In the TWG group, *Anaerocolumna* and *Exiguobacterium* were significantly enriched. *Anaerocolumna*, an anaerobic fermentative bacterium involved in the degradation of organic matter and energy conversion, was enriched under exposure to wildfire ash, indicating a shift toward a more anaerobic gut microenvironment that may help tadpoles maintain energy homeostasis under exogenous stress (Sparagon et al., 2022). *Exiguobacterium*, a facultative anaerobe tolerant to salt, alkali, and temperature, is often found in extreme environments and polluted waters. Its enrichment in the TWG group may be linked to increased gut ionic strength and microenvironmental changes from wildfire ash input (Xiao et al., 2024).

In the present study, LEfSe analysis indicated that different types of ash led to the enrichment of specific genera in the gut microbiota of adult *R. dybowskii*. The FCG group was significantly enriched in *Rhodoferax* and *Romboutsia*. *Rhodoferax*, with its flexible metabolic pathways and involvement in carbon cycling, provides energy and supports environmental adaptation in the host (Chen et al., 2025). *Romboutsia* degrades complex carbohydrates and synthesizes short-chain fatty acids, thereby maintaining intestinal barrier integrity and regulating inflammatory responses, making it a key marker of healthy intestinal microecology (Fan et al., 2024). In the FSG group, *Carnobacterium* and *Faecalibacterium* were significantly enriched. *Carnobacterium* can tolerate high salinity and alkalinity. It lowers intestinal pH through lactic acid fermentation, thereby inhibiting pathogenic bacteria and promoting intestinal mucosal repair, while enhancing the host's tolerance to harmful substances in straw ash (Ishikawa et al., 2003; Kaktcham et al., 2018). *Faecalibacterium* provides energy for intestinal epithelial cells, alleviates inflammatory responses, enhances antioxidant defense, and aids the host in maintaining microecological homeostasis and repairing damage under straw ash stress (Han et al., 2024). In the FWG group, *Cetobacterium, Dubosiella*, and *Pseudomonas* were enriched. *Cetobacterium* is effective at synthesizing vitamin B_12_ and promoting protein digestion, and it shows strong tolerance to high concentrations of heavy metals and alkaline conditions in ash (Xie et al., 2022; Zhang et al., 2022). *Dubosiella* assists in intestinal barrier repair by producing short-chain fatty acids and regulating metabolism (Zhu et al., 2022). *Pseudomonas* can degrade various harmful substances in ash and form biofilms, which provide detoxification defense for the host and help inhibit colonization by pathogenic bacteria (Li et al., 2020; Rajendran et al., 2022). The enrichment process may, to some extent, compensate for the ecological niches left by the loss of microbial diversity. However, persistent environmental pressure may cause the gut microbial community structure to shift toward simplification, thereby increasing ecosystem vulnerability (Huang et al., 2020; Pereira and Berry, 2017).

### 4.4 Function

We observed significant differences in the bacterial phenotypes of gut microbiota, including Gram-positive, Potentially-pathogenic, and Stress-tolerant traits, during tadpole metamorphosis into frogs in response to different ash treatment conditions. However, differences in bacterial phenotypes such as Aerobic, Anaerobic, Biofilm-forming, Facultatively-anaerobic, and Gram-negative were primarily evident in the tadpole stage. This indicated that substantial alterations in gut microbiota throughout the tadpole-to-frog transition were influenced by both developmental phases and environmental factors such as ash treatment (Minich et al., 2022). These findings emphasize the crucial roles of biological development and environmental variables in influencing the gut microbiota (Tong et al., 2020a). This was likely due to developmental phases, environmental stress from ash treatment, and alterations in gut flora and the immune system (Santos et al., 2023a,b). Since tadpoles primarily live in water and filter-feed on microorganisms, their gut structure and function were adapted to a predominantly Anaerobic environment (Xie et al., 2020). Frogs are adapted to both terrestrial and aquatic environments, requiring adjustments in their gut ecosystems to support Aerobic, Anaerobic, and Facultatively-anaerobic bacteria (Zhang et al., 2020). Different ash treatments could have resulted in variations in trace elements and chemical substances, which imposed selective pressure favoring Stress-tolerant gut microbiota (Evariste et al., 2021). Specifically, Gram-negative bacteria, due to their stronger environmental adaptability, may have had an advantage under adverse conditions (Rebollar et al., 2016). Biofilm-formation offered extra protection against environmental stress, enhancing the adaptability of microbial populations in the evolving gut environment (Wang et al., 2023). The tadpole stage represented a critical period for gut microbiota formation and adjustment, during which external environmental changes had a significant impact on the microbiota (Santos et al., 2023a,b). In contrast, frogs possess a mature immune system capable of effectively regulating gut microbiota composition and resisting Potentially-pathogenic organisms (Tong et al., 2023b; Zhang and Gallo, 2016).

### 4.5 Significance

Given the increasing prevalence of global warming, which exacerbates extreme weather conditions and wildfires, our findings are not only timely but also imperative for understanding the cascading effects on vulnerable species and ecosystems (Rajput et al., 2023; Romasanta et al., 2017). The research provides a detailed analysis of how the practice of burning rice straw (over 1 billion tons each year), a standard agricultural method, affects amphibian species (Ren et al., 2019). This aspect is crucial, as it bridges the gap between agricultural practices and wildlife conservation, illustrating how certain human activities can have unintended yet significant ecological impacts (Kakakhel et al., 2023; Williams-Subiza and Brand, 2021). The study also explored how different types of vegetation, represented by the various ashes, influence the gut microbiota of amphibians at various stages of their life cycle (Santos et al., 2023b). This understanding is vital for assessing how ecosystem changes—whether driven by wildfires or human activities—can have cascading effects on native species (Kakakhel et al., 2023; Williams-Subiza and Brand, 2021). Although this study was conducted in a controlled laboratory environment, the findings may also offer insights into broader ecological processes and the challenges associated with global warming and changing human activities (Rajput et al., 2023; Romasanta et al., 2017). The significant differences in microbial diversity between laboratory and natural environments highlight the profound impact of experimental conditions on research outcomes. In this rigorously controlled laboratory investigation, we observed a predominance of Firmicutes within the skin microbiota of frogs, which markedly diverged from our earlier field-based findings, where Bacteroidetes dominated the microbiota during winter hibernation (Tong et al., 2020b). This variation underscores the significant influence of experimental conditions on microbial diversity. Therefore, applying laboratory data to natural environments requires careful consideration (Punj et al., 2023). Given that data on ash concentrations in natural habitats remain limited, inferences regarding the susceptibility of wild populations should be interpreted with caution.

## 5 Conclusions

Our study provides the first characterization of the dynamic responses of gut microbiota to wildfire and agricultural (rice straw) ash exposure in *R. dybowskii* across both tadpole and adult stages, revealing distinct stage-specific sensitivities and alterations in microbiota composition. Wildfire ash demonstrated greater toxicity, significantly reducing tadpole survival and producing more pronounced disruptions of gut microbial community structure compared to rice straw ash. Adult frogs exhibited greater tolerance. However, they exhibited notable compositional shifts, particularly under exposure to wildfire ash. Functional analysis revealed that key bacterial taxa and predicted phenotypes (e.g., stress tolerance and pathogenic potential) were closely linked to ash type and developmental stage. Collectively, our findings demonstrate the intricate relationship between ash-derived environmental stressors, gut microbiota structure, and amphibian health, emphasizing the critical need for ash-targeted mitigation strategies and microbiota-guided conservation efforts to protect amphibian populations in fire-disturbed and agricultural landscapes. This study was conducted under controlled laboratory conditions, which may not fully capture the complexity and diversity of natural environments. Future research should prioritize long-term field studies to evaluate the lasting effects of ash exposure and potential interactions with other environmental pollutants on amphibian gut microbiota.

## Data Availability

The datasets presented in this study can be found in online repositories. The names of the repository/repositories and accession number(s) can be found in the article/Supplementary material.
