# Supplementary material for: Dynamic responses of gut microbiota to agricultural and wildfire ash: insights from different amphibian developmental stages
Source: Front Microbiol. 2025 Aug 13;16:1598446. doi: 10.3389/fmicb.2025.1598446 (PMC12380842; doi:10.3389/fmicb.2025.1598446)
Supplement: Supplementary file 1 [file Data_Sheet_1.docx]

**Dynamic Responses of Gut Microbiota to Agricultural and Wildfire Ash: Insights from Different Amphibian Developmental Stages**

Qing Tong^a, b^, Ming-da Xu^a^, Qiu-ru Fan^a^, Yue-liang Pan^a^, Xin-zhou Long^a^, Wen-jing Dong^a^, Li-yong Cui^b^, Zhi-wen Luo^a*^

^a^School of Biology and Agriculture, Jiamusi University, Jiamusi, 154007, China

^b^Jiamusi Branch of Heilongjiang Academy of Forestry Sciences, Jiamusi, 154002, China

**^*^Corresponding author**

Zhi-wen Luo (jmslzw@126.com)

Tel.: +86-454-5602255

Fax: +86-454-5602255

Word count: 6500

Number of figures: 5

**
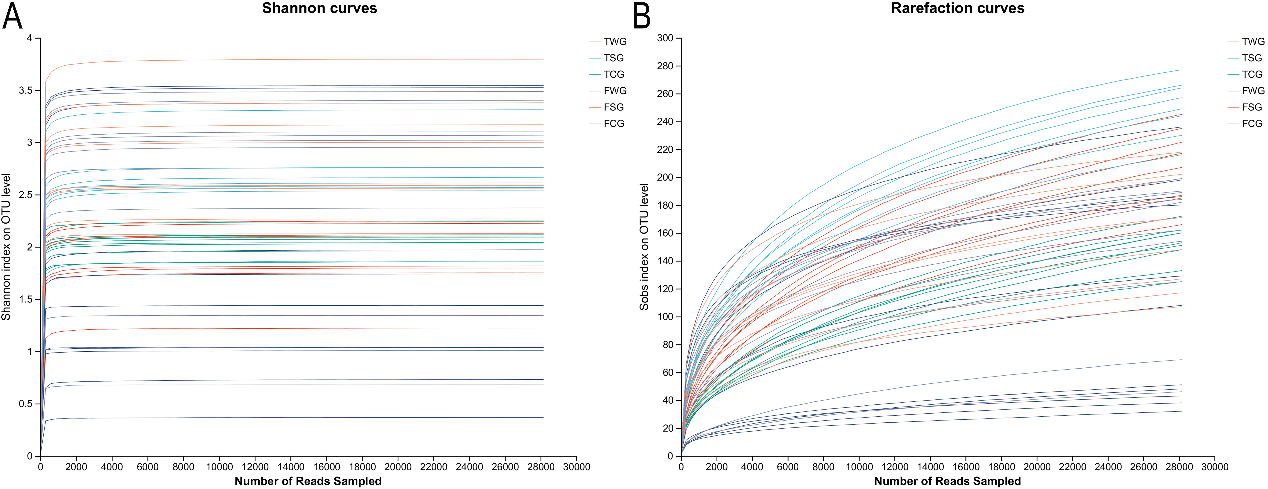
**

**Figure S1** Analyzing shannon indices and rarefaction

Provides a quantitative assessment of microbial diversity and species richness in the gut microbiota of tadpoles and frogs subjected to different treatments, utilizing Shannon curves (A) and rarefaction curves (B). The Shannon and Rarefaction curves visually represent the correlation between sequencing depth and detected OTUs, demonstrating microbial diversity in samples.


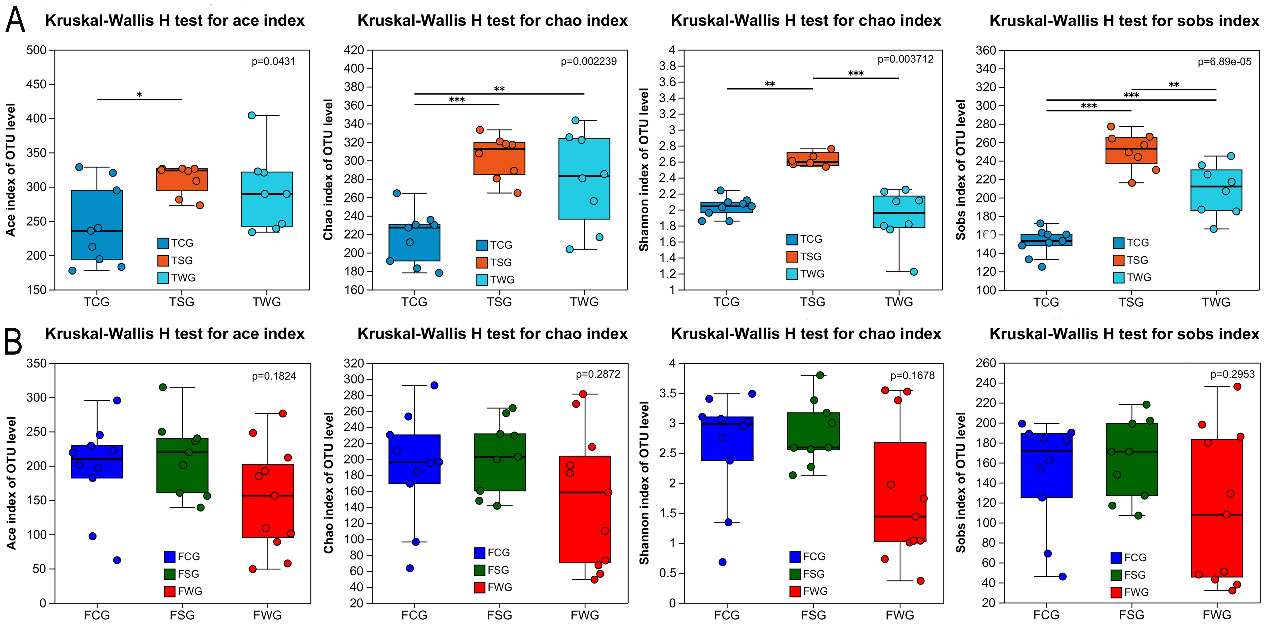


**Figure S2** Comparison of alpha diversity indices (Ace, Chao, Shannon, and Sobs) across control, straw ash and wildfire ash groups at adult and tadpole stages of gut microbiota

The Kruskal-Wallis H test or Tukey-Kramer post-hoc test was used to compare differences in alpha diversity indices (Ace, Chao, Shannon, and Sobs) across different groups (TCG, TSG, TWG, FCG, FSG, and FWG). (A) The differences in the tadpole stage (TCG, TSG, and TWG groups) are shown. (B) The comparison of groups in the adult stage (FCG, FSG, and FWG groups) is illustrated. Each boxplot shows the interquartile range, median, and outliers (marked as circles). Significance levels were indicated as *, **, and *** for 0.01 < *P* < 0.05, 0.001 < *P <* 0.01, and *P* < 0.001, respectively.


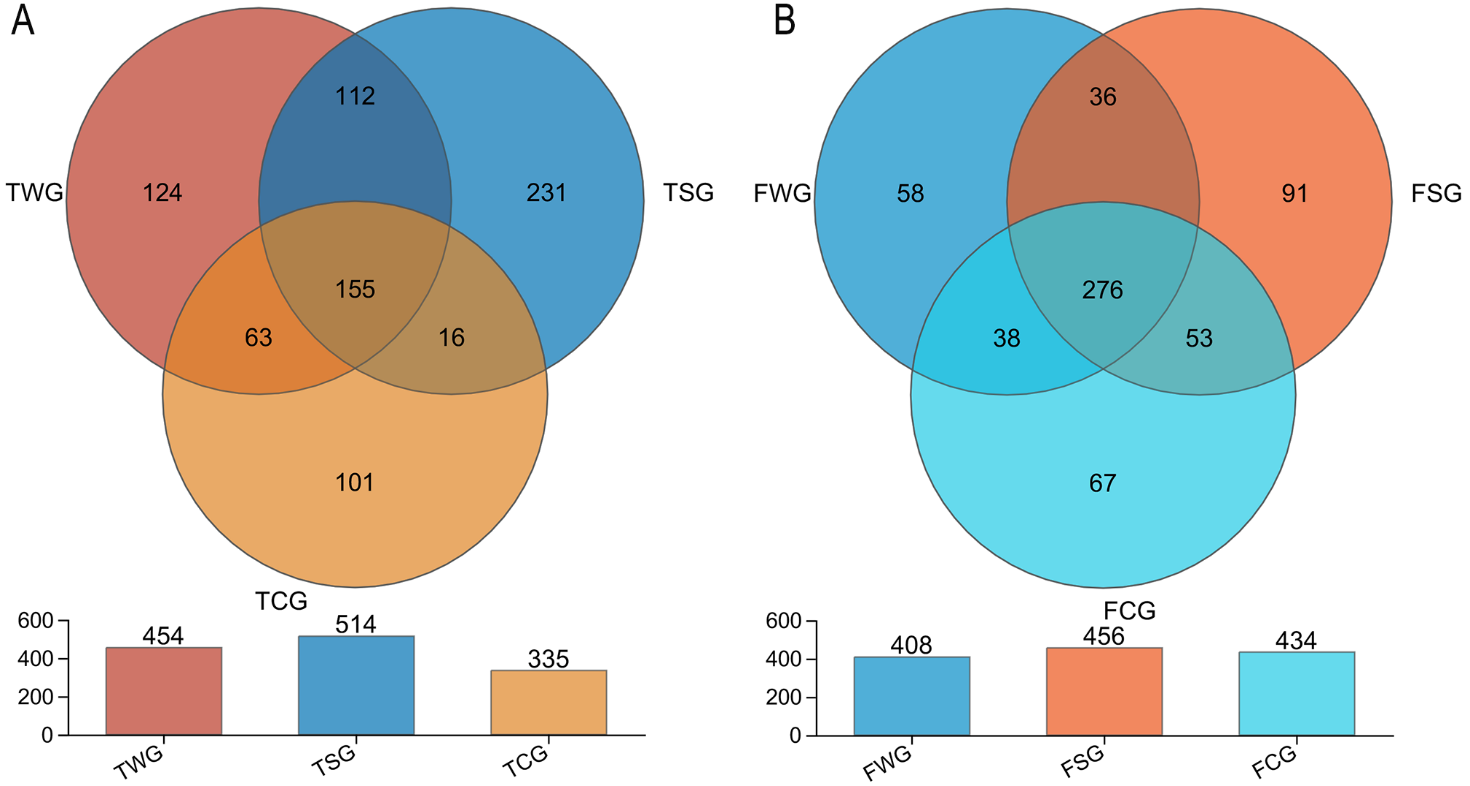


**Figure S3** Venn diagrams illustrating shared bacterial OTUs gut microbiota across different ashes treatment group

Numbers in the triple overlap Venn diagram show common OTUs across three groups, whereas neighboring figures represent distinct OTUs. Each group is represented by a distinctively colored circle. The bar graphs below represent the total number of OTUs detected in each category.


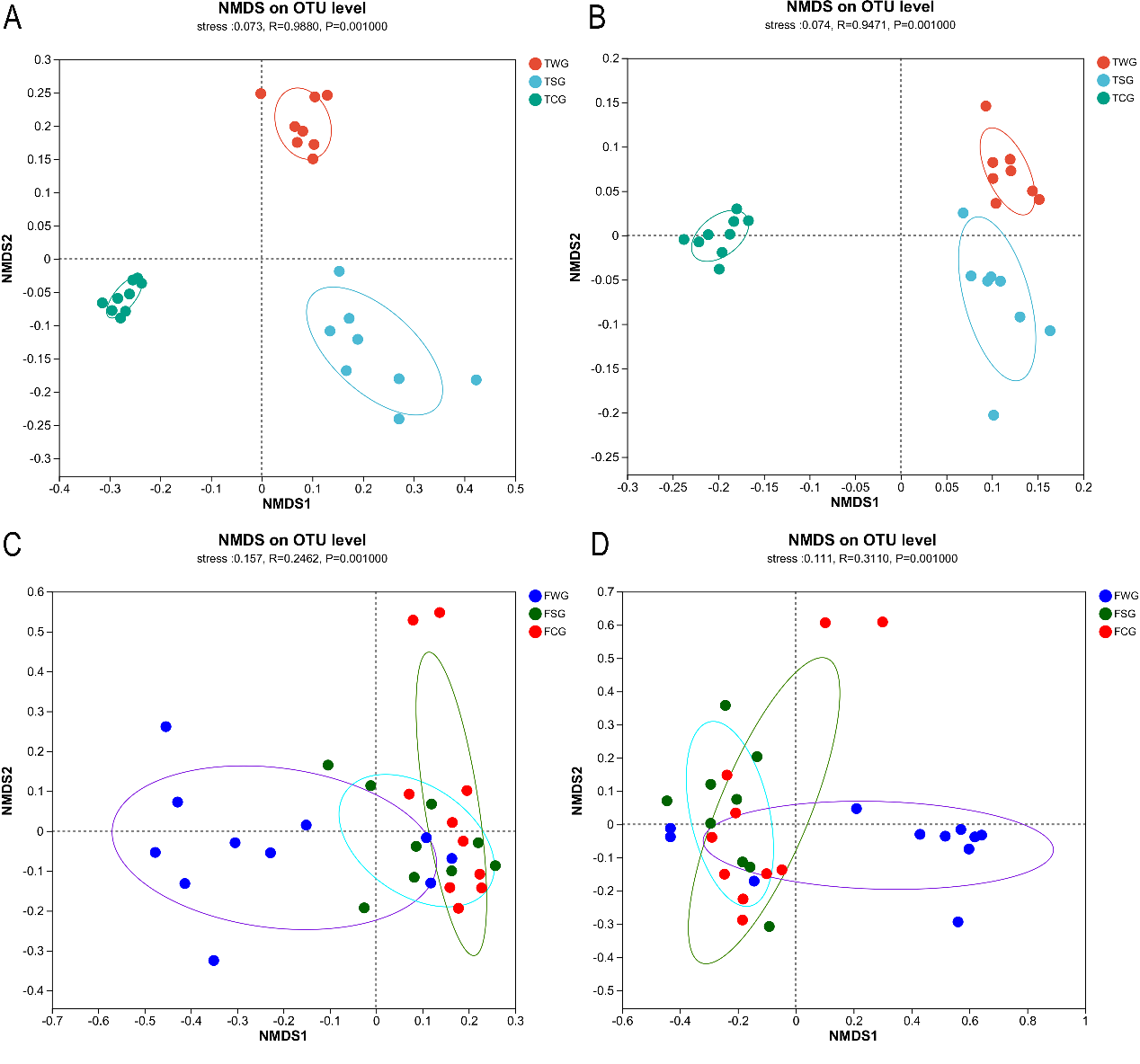


**Figure S4** Non-metric multidimensional scaling (NMDS) analysis the influences of rice straw and wildfire ash on the gut microbiota diversity of adults and tadpoles

The NMDS plot illustrates group differentiation patterns using Bray-Curtis (A and C) and weighted UniFrac (B and D) distances. Every point depicts a sample’s gut microbiota different groups and spatial proximity reflects similarity.


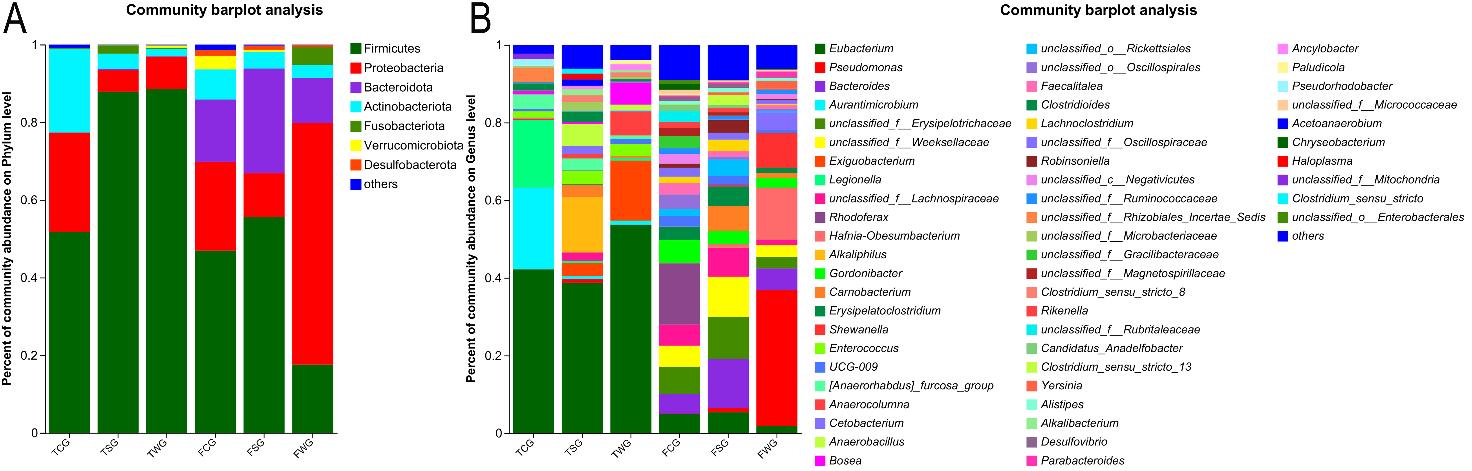


**Figure S5** Relative abundances of gut microbiota composition at treatment groups

Showing the relative abundance of different bacterial phyla (A) and genera (B) in gut microbiota samples. Each bar represents a specific phylum (or genus), with the height indicating its relative abundance. Significant differences in gut microbiota composition across gut samples. Distinct colors are used to represent different groups.


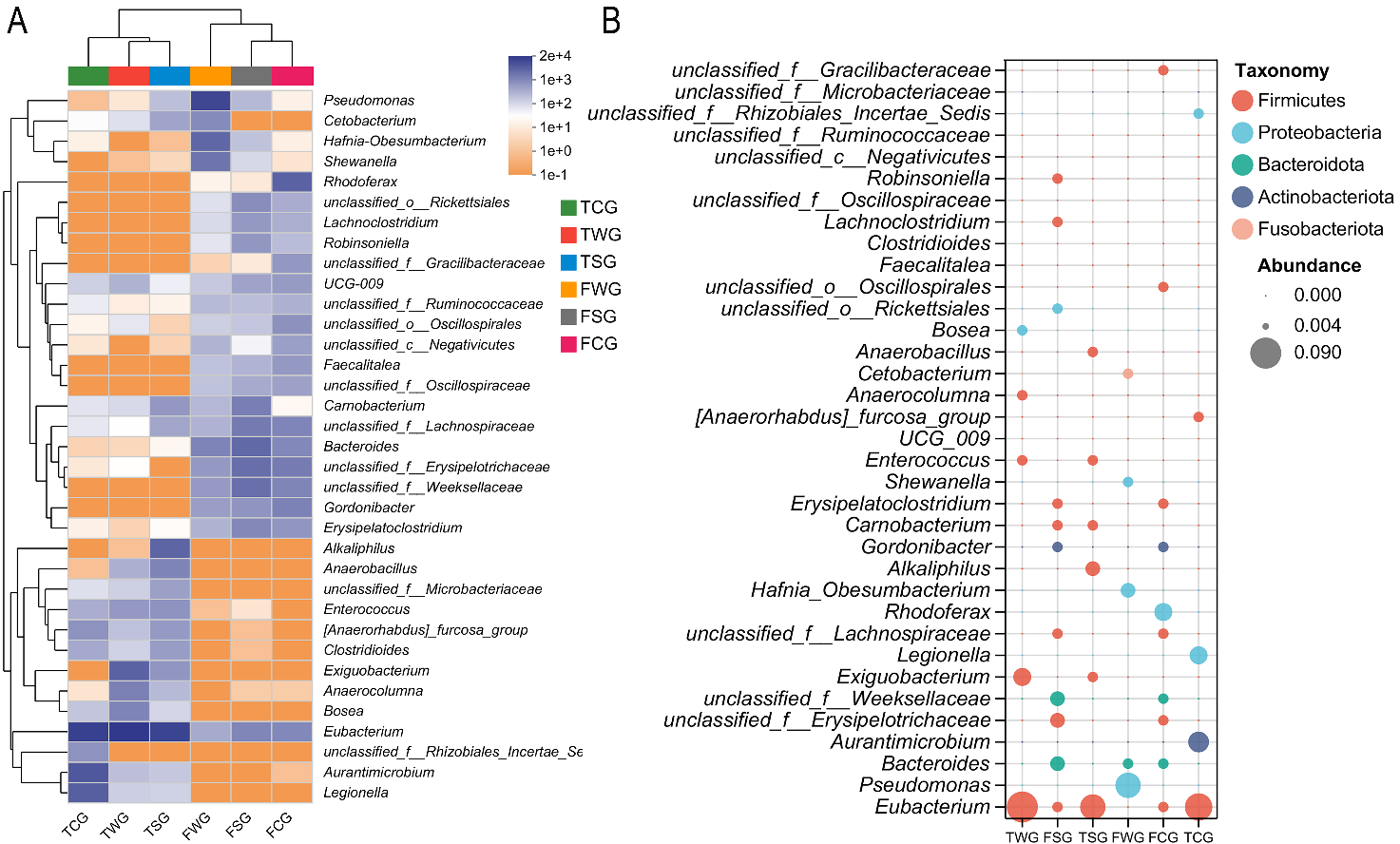


**Figure S6** Heatmap depicting genus-level variations across different ashes treatment groups

Using Bray-Curtis distances and the average-linkage method, with each bar or column representing an individual specimen. Relative abundance data are shown as log-transformed values (A). A green-to-red color scale is used to visualize these values, where green indicates lower abundance and red indicates higher abundance (B).


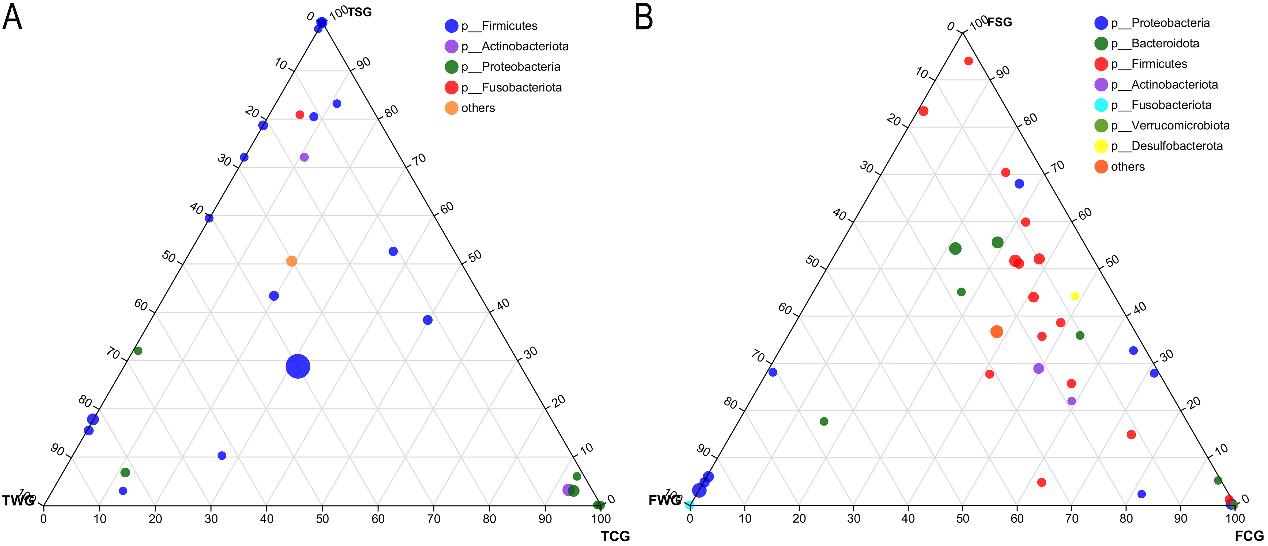


**Figure S7** Differences in the distribution of OTU and phyla across various compartments.

Each OTU was represented as a circle on a ternary plot that categorized them by compartment specificity, with circle size, color, and position indicating relative abundance, bacterial phylum, and compartment affinity, respectively.
